# How prepared is Africa to face COVID-19?

**DOI:** 10.11604/pamj.supp.2020.35.2.22665

**Published:** 2020-04-08

**Authors:** Raoul Emeric Guetiya Wadoum, Andrew Clarke

**Affiliations:** 1 Department of Public Health, Microbiology and Immunology, Ernest Bai Koroma, University of Science and Technology, Makeni, Sierra Leone; 2 Global Programs Division, Save the Children UK, London, UK

**Keywords:** Infectious Diseases, Coronaviruses, COVID-19, SARS-CoV-2, Pandemic, Public health

## Abstract

The epidemic of Coronavirus disease 2019 (COVID-19) in China caused by the Severe Acute Respiratory Syndrome Coronavirus 2 (SARS-CoV-2) has become a global concern and subsequently labeled a pandemic by the World Health Organization on March 11th. As the world mobilizes to contain the COVID-19, scientists and public health experts are increasingly alarmed about the potentially catastrophic effects of an outbreak in Africa. The establishment of Africa Centres for Disease Control and Prevention by the Africa Union in 2017 was an unprecedented move toward strengthening national responses, so far enabling all fifty member states with confirmed cases of COVID-19 to adequately respond, break chains of transmission and effectively contain the spread of SARS-CoV-2. We enter an uncertain and challenging period that may severely test the preparedness, organizational resource and resilience of African states and the fabric of their societies. However, we speculate that the fear associated with COVID-19 may also lead to some of the long-standing messages about simple measures to reduce the spread, such as hand washing, finally becoming absorbed and more universally adopted by health workers and the public. Is it possible that regardless of the terrible threat posed by SARS-CoV-2, the increased adoption of these health protection measures may result in a reduction in the spread of other infectious diseases?

## Commentary

Coronavirus disease 2019 (COVID-19), the disease caused by the Severe Acute Respiratory Syndrome Coronavirus 2 (SARS-CoV-2), is spreading globally with over 206 countries and territories affected 1,051,635 confirmed cases, and 56,985 deaths recorded as of 4th April 2020 [1]. Following rapid expansion, the World Health Organization (WHO) declared COVID-19 a pandemic on March 11th. As the world mobilizes to support the WHO declaration of COVID-19 a Public Health Emergency, and subsequently struggles to manage the rapid emergence of the infection, scientists and public health experts are raising a serious alarm about the catastrophic effects that an outbreak in Africa could have, given that public health systems throughout Africa are weak and most of the continent lacks the global health security capabilities and social protection infrastructure necessary to adequately respond and manage outbreaks and the cascade of subsequent effects on society, in particular the most vulnerable sections. History has taught us how new pathogens emerge randomly and cause outbreaks with global impact and the celebration of the 100th anniversary of the Spanish Flu pandemic in 2018 that infected 500 million people, resulting in over 100 million death around the world was a painful reminder. Since then, the world has not seen a public health emergency of that scale, although the West African Ebola outbreak in 2014 was evidence that the planet remains vulnerable, and we are not aware of when and where the next global pandemic will occur.

The original situation in China became a global concern, with the immediate and future health of more than 7 billion people now at stake and the gains made over recent decades in development and prosperity now at risk of being undermined unless interstate coordination is strengthened to contain the epidemic. The United Nations (UN) Charter signed on 26 June 1945 in San Francisco and entered into force on 24 October 1945 laid the foundation for such collaboration. The UN is an intergovernmental organization tasked with maintaining international peace and security as well as the development of friendly relationships among nations. This mission is achieved by UN´s specialized agencies such as the Food and Agriculture Organization of the UN, the UN Educational, Scientific and Cultural Organization, the United Nations Children´s Fund, and the World Health Organization, among others [2]. In the Africa region, leaders expressed their desire and commitment to take a greater role and ownership for promoting unity, solidarity, cohesion and cooperation among the peoples of Africa by forming a Pan Africa institution, the Africa Union (AU). In the constitutive act of the AU, member states recognized the need for addressing the negative impact of diseases by adopting and implementing strategic policies to reduce the burden and improve public health in Africa [3]. Concerned by Africa´s increasing disease burden, the AU established health institutions to ensure that member states develop and sustainably manage their health sectors by putting in place programs to support the training of health professionals, public health preparedness, and adequate response to emergencies and disease epidemics in the continent. As a technical guide, the AU developed the Africa Health Strategy 2016-2030 aligned with the international health regulations and aimed to steer member States [4].

A stronger, more forward-looking interstate collaboration was achieved during the West Africa Ebola virus disease epidemic, where nations worldwide joined hands to support and contain the epidemic. In Africa, AU member states through the Peace and Security Council and the executive committee established the African Union Support to the Ebola Outbreak in West Africa (ASEOWA) tasked to assist the outbreak in Sierra Leone, Guinea and Liberia. Based on the achievement of ASEOWA, the Africa Centres for Disease Control and Prevention (Africa CDC) was established in 2017 as the first public health institute mandated to harmonize infectious diseases capacity building, surveillance, and response to emergencies among a group of independent countries [5,6]. With the fallout from Ebola serving as a powerful lesson, the Africa CDC activated straight away its Emergency Operations Centre (EOC) and its Incident Management System (IMS) for the COVID-19 outbreak on 27 January 2020 to step-up with health authorities of AU member states protocols and implement emergency preparedness procedures. Africa CDC initial action was the selection together with WHO of 13 member states as high priority based on the volume of travel from China including Algeria, Angola, Ivory Coast, the Democratic Republic of the Congo, Ethiopia, Ghana, Kenya, Mauritius, Nigeria, South Africa, Tanzania, Uganda, and Zambia. Subsequent activities implemented were the training of laboratory personnel across 27 countries and the distribution of test kits, infection prevention and control training for 22 countries, and Training-of-Trainers events on points-of-entry surveillance for 18 countries [7].

The Bill & Melinda Gates Foundation and the Skoll Foundation announced a commitment of $12 million to the Africa CDC to help African nations scale up preparedness for a potential spread of SARS-CoV-2. This includes funding for “technical support to implement the screening and treatment of suspected cases, laboratory confirmation of 2019-nCoV diagnoses, and the safe isolation and care of identified cases.” An additional donation of 5.4 million face masks, more than 1 million testing kits, 40,000 items of protective clothing and 60,000 sets of face shields by the Jack Ma and Alibaba Foundations has consolidated emergency response and preparedness actions towards implementation of the Africa joint continental strategy for COVID-19 led by the AU through Africa CDC [8,9]. COVID-19 outbreak represents Africa CDC´s second complex health challenge alongside the ongoing Ebola and measles outbreaks in DR Congo. However, the extraordinary commitment and intensive cross-continent coordination with sister organizations such as the US CDC, European CDC, China CDC, WHO, among others has enabled Africa CDC to adequately contain confirmed cases so far of COVID-19 disease reported in all African countries except in Sao Tome and Principe, Comoros, Lesotho, and South Sudan currently uninfected by COVID-19 [7].

How long this position can be maintained remains to be seen. The infectiousness, fatality rate, and transmission patterns of SARS-CoV-2 in Africa are uncertain and the current fatality rate appears to be approximately 2 percent. By comparison, the outbreaks of Ebola and measles still ongoing in DR Congo have, respectively, fatality rates of over 60 and over 90 percent. However, as we witness the rapid spread of SARS-CoV-2 across increasingly diverse and large parts of the world´s population, within countries with greater resource and organizational capacity to draw upon than most in Africa, and despite radical restrictions on population movement, we can only brace ourselves and demand extensive planning based on evidence and pre-mobilization of the resource. The secondary effects on society in the most affected countries, of extensive steps being taken to prevent further spread, are also become increasingly apparent. These include restrictions on movements, social isolation, not being able to work and earn, go to school, and for other public services to function, and will affect the poorest and least resilient in our communities hardest and quickest. It is to be hoped that the additional time our governments and populations have had to prepare and raise awareness, will translate into more managed outbreaks; but given the fragility of our health systems, it is hard to foresee how any level of a surge in demand for high-intensity health care will be accommodated.

While the virus has stretched the resources of industrialized nations in Europe and the US to breaking point, in a globalized world, the case needs to urgently be made for richer countries to look beyond their pressing domestic needs and pragmatically support the response in Africa as their own. In the midst of the uncertainly and deep concern about how African states will cope with epidemic SARS-CoV-2, there is perhaps one note of cautious optimism for life beyond the present. Fear about the virus is widespread, and it may be that some of the long-standing messages about simple measures to reduce the spread of disease, such as hand washing, may finally be absorbed and become more universally adopted by health workers and the public. Is it possible that regardless of the terrible threat posed by SARS-CoV-2, the increased adoption of these health protection measures may also result in a reduction in the spread of other infections that have blighted our populations for too long? The establishment of Africa CDC was an unprecedented move toward strengthening national responses enabling countries to adequately respond to a potential outbreak in Africa and identify threats early enough, respond effectively, while combating infectious diseases burden including HIV/AIDS, TB, and Malaria, among others. We are now in a period that will test the robustness of our capacity to respond in a way seldom seen before, and the willingness of richer nations to support us do so, in the interests of the global community.

## Competing interests

The authors declare no competing interests.
